# Factors related to intra-tendinous morphology of Achilles tendon in runners

**DOI:** 10.1371/journal.pone.0221183

**Published:** 2019-08-14

**Authors:** Kai-Yu Ho, Ari Baquet, Yu-Jen Chang, Lung-Chang Chien, Michelle Harty, Gregory Bashford, Kornelia Kulig

**Affiliations:** 1 Department of Physical Therapy, University of Nevada, Las Vegas, Las Vegas, Nevada, United States of America; 2 Division of Biokinesiology & Physical Therapy, University of Southern California, Los Angeles, California, United States of America; 3 Division of Physical Therapy, West Virginia University, Morgantown, West Virginia, United States of America; 4 Epidemology and Biostatistics, Department of Environmental and Occupational Health, School of Public Health, University of Nevada, Las Vegas, Nevada, United States of America; 5 Department of Biological Systems Engineering, University of Nebraska-Lincoln, Lincoln, Nebraska, United States of America; University of Pittsburgh, UNITED STATES

## Abstract

The purpose of this study was to determine and explore factors (age, sex, anthropometry, running and injury/pain history, tendon gross morphology, neovascularization, ankle range of motion, and ankle plantarflexor muscle endurance) related to intra-tendinous morphological alterations of the Achilles tendon in runners. An intra-tendinous morphological change was defined as collagen fiber disorganization detected by a low peak spatial frequency radius (PSFR) obtained from spatial frequency analysis (SFA) techniques in sonography. Ninety-one runners (53 males and 38 females; 37.9 ± 11.6 years) with 8.8 ± 7.3 years of running experience participated. Height, weight, and waist and hip circumferences were recorded. Participants completed a survey about running and injury/pain history and the Victorian Institute of Sport Assessment-Achilles (VISA-A) survey. Heel raise endurance and knee-to-wall composite dorsiflexion were assessed. Brightness-mode (B-mode) sonographic images were captured longitudinally and transversely on the Achilles tendon bilaterally. Sonographic images were analyzed for gross morphology (i.e., cross-sectional area [CSA]), neovascularization, and intra-tendinous morphology (i.e., PSFR) for each participant. The factors associated with altered intra-tendinous morphology of the Achilles tendon were analyzed using a generalized linear mixed model. Multivariate analyses revealed that male sex was significantly associated with a decreased PSFR. Additionally, male sex and the presence of current Achilles tendon pain were found to be significantly related to decreased PSFR using a univariate analysis. Our findings suggested that male sex and presence of current Achilles tendon pain were related to intra-tendinous morphological alterations in the Achilles tendon of runners.

## Introduction

Achilles tendinopathy is a common overuse disorder in runners[1, 2] related to repetitive overloading of the Achilles tendon. Achilles tendinopathy has been found to be up to ten times more prevalent in runners than in the general population[2], and is the most prevalent musculoskeletal injury reported by athletes participating in ultra-marathons [1]. Various pathological changes within the tendinopathic tendon have been documented, including increased tendon cross-sectional area (CSA)[3], increased tendon thickness[4], increased water content[5], neovascularization[6], elevated sulfated glycosaminoglycan content[7], and altered collagen orientation[5] and composition[8].

Ultrasound imaging of the Achilles tendon has been used to assist the diagnosis of Achilles tendinopathy[9]. The most common abnormalities in tendinopathic tendons observed in sonography include increases in CSA and thickness, presence of neovascularization, and/or presence of hypoechoic areas[9–11]. It should be noted that while these gross morphological measures reliably reflect tendon pathology[10, 11], little is known about the intra-tendinous morphological alterations of the Achilles tendon in a at-risk population (e.g., runners). Additionally, Doppler detection of neovascularization is sensitive only above certain blood flow velocities and can be affected by ankle dorsiflexion position[12], and the assessment of neovascularization and hypoechoicity can be somewhat subjective[13].

The ability to quantify intra-tendinous morphology changes, indicative of disorganization of tendon collagen fibers, may prove more sensitive than gross morphologic changes or human interpretation of greyscale images [14, 15]. Bashford et al.[14] developed analyses to assess the collagenous arrangement of tendons by assessing spatial frequency parameters. Spatial frequency analysis (SFA) estimates spectral parameters indicative of the organization of the collagenous arrangement, which is indirectly represented by the “speckle pattern”[4, 15] visible on ultrasound imaging. SFA operates in two dimensions and thus analyzes both axial and lateral spatial frequency components simultaneously. SFA is different than but complementary to spectrum analysis methods which operate on the raw radio-frequency data in the axial dimension (e.g. [16]). The underlying operation of SFA (Fourier transform) is rotationally invariant and thus less dependent on probe angle than axial-only analyses. Of the eight spectral parameters originally described [14], the peak spatial frequency radius (PSFR) has been most studied. A lower PSFR reflects greater collagenous disarray, which is one underlying structural phenomenon of degeneration[13]. On the contrary, a higher PSFR reflects a greater collagenous density and/or organization [14, 15]. Kulig and colleagues [15] found that healthy tendons exhibit a mean PSFR value of 2.07 mm^-1^, while the mean PSFR value of degenerated tendons is around 1.55 mm^-1^.

The existing literature reveals several factors related to Achilles tendinopathy in general and athletic populations, including male sex[17, 18], middle age[19], obesity[20], increased years of running[21], altered plantarflexor muscle performance[21–23], and decreased dorsiflexor flexibility[18, 24, 25]. However, how these factors contribute to intra-tendinous morphological alterations of the Achilles tendon in runners remains unclear. Thus, this study aimed to determine and explore factors (age, sex, anthropometry, running and injury/pain history, tendon gross morphology, neovascularization, ankle joint range of motion, and plantarflexor muscle endurance) related to intra-tendinous morphological alterations of the Achilles tendon in runners. An intra-tendinous morphological change was defined as collagen fiber disorganization detected by a low PSFR on sonography.

## Materials and methods

### Participants

The inclusion criteria were age over 18 years across any sex and running on average at least 9.7 km (6 miles) per week [26] for the past 3 months or for the majority of the training year. Participants were also required to have not completed a long-distance run (half marathon or marathon) within 3 days of study participation. Pregnant runners were excluded from the study. A relatively lower milage (6 mile per week) was chosen as a criterion in order to include runners with a wider level of experiences. Both symptomatic and asymptomatic individuals were included as we would like to explore the associations between clinical symptoms and PSFR. This study was performed at two different sites, and was approved by the Institutional Review Boards at the University of Southern California (IRB # HS-16-00272) and University of Nevada, Las Vegas (IRB # 902392). All participants provided written informed consent prior to data collection, per Institutional Review Board protocols. Ninety-one participants (53 males and 38 females) were included in this study.

### Procedures

Data collection sessions occurred in two consecutive phases. In the first phase, the following data were acquired by either A.B. or M.H.: running and injury/pain history, anthropometrics, and selected clinical tests. Running and injury/pain history were collected via a questionnaire designed for this study, and participants completed the Victorian Institute of Sport Assessment—Achilles (VISA-A), a self-administered questionnaire on Achilles tendon-related function and participation[27]. Anthropometric measures consisted of height, weight, hip circumference, and waist circumference. Clinical tests consisted of a knee-to-wall composite dorsiflexion test (a weight-bearing lunge test of composite ankle dorsiflexion range of motion, as described by Hoch and Mckeon[28]) and a heel raise endurance test[29] performed off a step through full active range of motion into elevation and lowering at the rate of 1 repetion per second. The heel raise endurance test was chosen because it is a commonly-utilized test to represent the overall ankle plantarflexor performance during weight-bearing[29]. Both knee-to-wall compositie dorsiflexion test and heel raise endurance test were examined on both limbs.

In the second phase, one of three sonographers (A.B., M.H., and Y.C.) performed palpation of the tendon’s full length at medial, lateral, anterior, and posterior aspects; and acquired brightness-mode (B-mode) ultrasound images of bilateral Achilles tendons using a portable ultrasound imaging scanner (GE LOGIQ-e, GE Healthcare, Milwaukee, WI, USA). Achilles tendons were scanned while the participant lay prone with the knee in an extended position and the ankle passively maintained in neutral position, as described and visually represented by Kulig et al.[15] A linear array transducer (GE 12L-RS, bandwidth 5-13MHz, width 38.4 mm, GE Healthcare, Milwaukee, WI, USA) was used with the musculoskeletal preset at a depth of 2 cm (Bashford et al., 2008). Images were captured in three locations longitudinally, centered at 0, 2, and 4 cm from the superior aspect of the calcaneus. A cross-sectional transverse image was also captured at the 2 cm location to quantify CSA of the tendon. Next, tendons were assessed at all 3 longitudinal locations for color Doppler signal, and images were captured if neovascularization was observed. Image capture was carefully performed to ensure optimal image quality and minimize movement artifacts.

In all, the variables collected for this study were: age, sex, anthropometry (i.e., body mass index [BMI] and waist-to-hip ratio), running and Achilles tenodon injury/pain history (i.e., years of running, presence of current Achilles tendon pain, history of Achilles tendon pain, and VISA-A score), tendon gross morphology (i.e., CSA), neovascularization, ankle range of motion (i.e., knee-to-wall composite dorsiflexion), and heel raise endurance.

### Image analysis

Images were stored on the ultrasound unit in JPEG format. JPEG files were analyzed for CSA using the public-domain software ImageJ (National Institute of Health, v1.366, Bethesda, MD, USA). To reduce user error in selecting the outline of the tendon, the outcomes of 3 consecutive analyses were averaged to determine each tendon’s CSA and PSFR.

**Fig 1:**
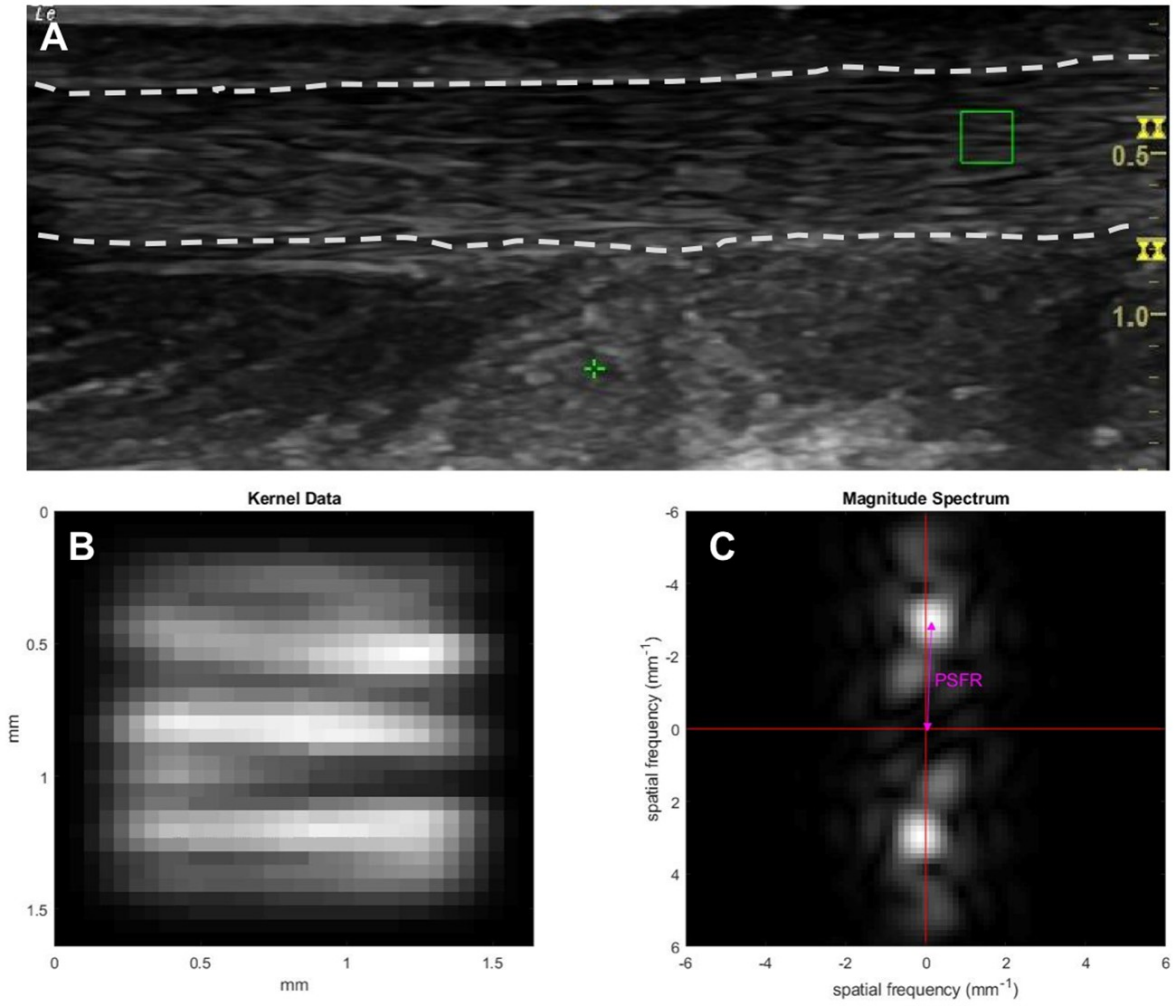
Frequency analyses of an Achilles tendon ultrasound image. (A) Original B-mode image with a sample kernel (green rectangle); (B) the zoomed data and spectrum of the kernel; (C) corresponding frequency spectrum of the selected kernel with a double arrow denoting peak spatial frequency radius. Area between the two dashed lines on the B-mode image is the Achilles tendon.

To perform intra-tendinous morphological assessment, images were analyzed with a custom MATLAB script (The MathWorks, Natick, MA, USA)[14]. A region of interest (ROI) was selected within the image situated 2 cm from the distal tendon insertion (Fig 1A). Although tendon length varies between participants, this selection standardized the use of a set region of mid-substance tendon proximal to the distal bony insertion. The ROI was selected to include the maximum visible tendon and peritenon area, and to exclude the distal and proximal image boundaries, in order to reduce error due to the distal and proximal tendon curvature. Within the ROI, 32 x 32 pixel kernels were extracted (representing 2 mm by 2 mm areas) and all possible kernels within the ROI were processed (Fig 1B). A second custom MATLAB script was used to perform a two-dimensional (2D) Fast Fourier Transform (FFT) of each kernel, from which the PSFR was extracted. The PSFR is calculated by finding the spatial frequency with the maximum amplitude in the spectrum (Fig 1C). This value is the same as the spectral “distance” from the spectrum origin to the maximum value and has thus been termed the “radius.” The intra-tendinous morphology analysis process has previously been described in full[14].

### Imaging reliability

To assess intra-rater reliability of image acquisition, 3 sonographers (A.B., M.H., and Y.C.) performed repeated data collection on 5 participants, separated by 1 week. To assess image analysis reliability of the two responsible investigators (A.B. and M.H.), five images were randomly chosen and the PSFR was measured on two separate days at least a week apart. As Y.C. only participated in image acquisition and A.B. analyzed the data collected by Y.C., image acquisition reliability was acquired by A.B., M.H., and Y.C and image processing reliability was acquired by A.B. and M.H. Intra-class correlation coefficient (ICC_(2,1)_) and standard error of measurement (SEM) were used to determine intra-rater reliability of image acquisition and image analysis. ICC analysis was chosen as it reflects both degree of correlation and agreement between data measured by each rater across 2 sessions, whereas other measures (e.g. paired t test and Bland-Altman plot, Pearson correlation coefficient) account for either agreement or correlation only [30]. The SEM was estimated by multiplying the standard deviation by √ 1 minus the reliability coefficient. Individual intra-rater reliability for PSFR was as follows: A.B. (acquisition: ICC = 0.868 and SEM = 0.019 mm^-1^; analysis: ICC = 0.958 and SEM = 0.014 mm^-1^), M.H. (acquisition ICC = 0.971 and SEM = 0.018 mm^-1^; analysis ICC = 0.977 and SEM = 0.014 mm^-1^), and Y.C. (acquisition: ICC = 0.920 and SEM = 0.024 mm^-1^).

### Statistical analysis

Summary statistics were performed on individual characteristics and related measurements in terms of mean, standard deviation, minimum, the 1^st^ and 3^rd^ quartiles, median, and maximum. Binary measurements (sex, presence of current Achilles tendon pain, history of Achilles tendon pain, and neovascularization) were summarized in frequencies and percentages. A correlation matrix was constructed in terms of the Pearson correlation coefficient for all continuous measurements, the Spearman correlation coefficient for all binary measurements, and the point-biserial correlation coefficient between each continuous measurement and each binary measurement. In the correlation matrices, variables with different measurements in two legs were regarded as separate and independent data points.

We used a general linear mixed model to evaluate the association between altered intra-tendinous morphology (i.e., PSFR) of bilateral Achilles tendons and related individual characteristics (age, sex, BMI, waist-to-hip ratio, years of running, presence of current Achilles tendon pain, history of Achilles tendon pain, and VISA-A score), and the repeated measurements for the two legs (heel raise endurance, knee-to-wall composite dorsiflexion, CSA, and neovascularization). The general linear mixed model was chosen because it takes into consideration the variance component between the PSFR and those predictors measured bilaterally in each individual. A univariate analysis was carried out for PSFR predicted by each predictor. For a multivariate analysis, all predictors were considered in the same model to ensure that the literature-documented risk factors of Achilles tendon degeneration were taken into consideration simutaneously. We adopted the variance inflation factor to diagnose multicolliearity in the multiavaraite model. Statistical analyses were performed with SAS v9.3 (SAS Institute Inc., Cary, NC, USA), and the significance level was set as p < 0.05.

## Results

The summary descriptive statistics of PSFR and the related predictors from the 91 participants shown in Table 1 revealed the following characteristics: 37.9 years-old average age, 24.1 average BMI, 0.84 average waist-to-hip ratio, and a higher percentage of males (58.2%). In addition, participants had an average of 8.8 years in running history, and 64.8% of participants had a history of pain. The average VISA-A score was 88, while the range was wide from 14 to 100 points. Both legs had an even proportion of the presence of current Achilles tendon pain. The correlation matrix shown in Table 2 indicated that the associations across all predictors were small, and only a few predictors were moderately correlated, such as BMI, waist-to-hip ratio, and sex as well as the presence of current Achilles tendon pain and history of Achilles tendon pain. Given that the predictors were not highly inter-correlated, the chance of multicollinearity was expected to be low in the multivariate analysis of general linear mixed model when all predictors were considered.

**Table 1:** Descriptive statistics of peak spatial frequency radius and related predictors.

Variable		Mean	SD	Min	Q1	Median	Q3	Max
Age		37.9	11.6	19.0	28.0	35.0	48.0	63.0
BMI		24.1	2.9	17.9	22.1	23.8	25.9	32.8
Waist-to-hip		0.84	0.06	0.71	0.80	0.84	0.88	0.98
Year running		8.8	7.3	0.5	4.0	6.0	11.0	30.0
VISA-A		88.0	17.6	14.0	82.0	96.0	100.0	100.0
CSA (mm^2^)	Left	59.8	20.2	30.0	48.2	54.7	66.0	128.4
	Right	58.4	17.9	22.7	47.1	54.0	65.5	127.1
Knee-to-wall (cm)	Left	10.7	4.5	2.0	7.5	10.8	13.0	25.0
	Right	10.8	4.1	0.0	7.5	10.8	13.5	21.0
Heel raise	Left	18.1	8.2	2.0	13.0	17.0	23.0	45.0
	Right	17.6	7.8	2.0	12.0	16.0	22.0	48.0
PSFR (mm^-1^)	Left	1.99	0.17	1.59	1.83	1.95	2.07	2.34
	Right	1.96	0.16	1.58	1.81	1.94	2.03	2.31
		**N**	**%**					
Sex	Male	53	58.2					
	Female	38	41.8					
Pain history	Yes	59 (35M; 22F)	64.8					
	No	32 (18M; 14F)	35.2					
		**Left** **N**	**%**	**Right** **N**	**%**			
Current pain	Yes	20	22.0	20	22.0			
	No	71	78.0	71	78.0			
Neovascularization	Yes	9	9.9	5	5.5			
	No	82	90.1	86	94.5			

Abbreviations: SD = standard deviation; Q1 = 1^st^ quartile; Q3 = 3^rd^ quartile; BMI = body mass index; Waist-to-hip = Waist-to-hip ratio; Year running = years of running; VISA-A = Victorian Institute of Sport Assessment—Achilles; CSA = cross-sectional area; Knee-to-wall = knee-to-wall composite dorsiflexion; Heel raise = Heel raise endurance; PSFR = Peak spatial frequency radius; Pain history = history of Achilles tendon pain; Current pain = Presence of current Achilles tendon pain; M = males; F = females.

**Table 2:** Correlation coefficients of peak spatial frequency radius and related predictors.

**Variable**	**PSFR**	**Age**	**Sex**	**BMI**	**Waist-to-hip**	**Year running**	**Current pain**	**Pain history**	**VISA-A**	**CSA**	**Neovascu-lization**	**Knee-to-wall**	**Heel raise**
**PSFR**	1.00	-0.04	-0.24*	-0.07	-0.15	-0.04	-0.18*	0.01	0.09	-0.10	0.07	0.09	-0.04
**Age**		1.00	0.14	0.09	0.33*	0.38*	0.08	0.16	-0.28*	0.03	0.28*	-0.28*	0.07
**Sex**^‡^			1.00	0.41*	0.56*	-0.18	0.15*	0.08	-0.14	0.28*	0.12	-0.12	0.17*
**BMI**				1.00	0.51*	-0.08	0.05	-0.14	-0.19*	0.09	0.27*	-0.26*	-0.08
**Waist-to-hip**					1.00	0.17*	0.07	0.04	-0.26*	0.13	0.19*	-0.19*	-0.11
**Year running**						1.00	-0.00	0.03	0.07	-0.03	-0.07	-0.01	-0.13
**Current pain**^‡^							1.00	0.47*	-0.38*	-0.04	0.07	-0.18*	-0.10
**Pain history**^‡^								1.00	-0.39*	0.07	0.08	-0.03	0.02
**VISA-A**									1.00	0.03	-0.21*	0.31*	0.04
**CSA**^†^										1.0	0.14	0.14	-0.06
**Neovascu-lization**^‡^											1.00	-0.16*	0.04
**Knee-to-wall**^†^												1.00	-0.15*
**Heel raise**^†^													1.00

^**†**^ The correlations were measured regardless of left and right legs in cross-sectional area, knee-to-wall composite dorsiflexion, and heel raise endurance.

^**‡**^ The correlations among binary sex, presence of current Achilles tendon pain, history of Achilles tendon pain, and neovasculization were measured by Spearman’s correlation. The correlations between the three binary variables and the other continuous variables were measured by point-biserial correlation.

* indicates a significant association (p < 0.05)

Abbreviations: PSFR = peak spatial frequency radius; BMI = body mass index; Waist-to-hip = waist-to-hip ratio; Year running = years of running; Current pain = presence of current Achilles tendon pain; Pain history = history of Achilles tendon pain; VISA-A = Victorian Institute of Sport Assessment—Achilles; CSA = cross-sectional area; Knee-to-wall = knee-to-wall composite dorsiflexion; Heel raise = heel raise endurance.

With respect to the findings of the general linear mixed model, the univariate analysis revealed that PSFR was significantly related to male sex and the presence of current Achilles tendon pain (Table 3). The univariate analysis showed that, compared to females, males had a significantly lower PSFR by 0.08 mm^-1^ (p < 0.01). Furthermore, participants with presence of current Achilles tendon pain in either leg had a significantly lower PSFR by 0.07 mm^-1^ (p = 0.02) than those without current Achilles tendon pain. When all predictors were considered in the model, the multivariate analyses showed that PSFR was still significantly related to male sex. Compared to females, males had a significantly lower PSFR by 0.10 mm^-1^ (p < 0.01) after adjusting for the other predictors (Table 3). The variation inflation factors varied from 1.17 to 2.19 across those predictors, confirming that no multicollinearity was detected in the multivariate model.

**Table 3:** Univariate and multivariate analyses of related predictors on peak spatial frequency radius.

Variable		Univariate Analysis			Multivariate Analysis		
		Estimate	95% confidence interval	p value	Estimate	95% confidence interval	p value
Age		-0.01	(-0.00, 0.00)	0.60	-0.00	(-0.00, 0.00)	0.46
Sex	Male	-0.08	(-0.13, -0.03)	**<0.01**^†^	-0.10	(-0.17, -0.04)	**<0.01**^†^
	Female	Reference			Reference		
BMI		-0.00	(-0.01, 0.00)	0.37	0.00	(-0.01, 0.01)	1.00
Waist-to-hip		-0.42	(-0.42 0.02)	0.06	0.20	(-0.42, 0.82)	0.52
Year running		-0.00	(-0.00, 0.00)	0.56	-0.00	(-0.00, 0.00)	0.66
Current pain	Yes	-0.07	(-0.13, -0.01)	**0.02**^†^	-0.05	(-0.12, 0.02)	0.15
	No	Reference			Reference		
Pain history	Yes	0.01	(-0.04, 0.06)	0.81	0.03	(-0.03, 0.09)	0.33
	No	Reference			Reference		
VISA-A		0.00	(-0.00, 0.00)	0.22	0.00	(-0.00, 0.00)	0.33
CSA		-0.00	(-0.00, 0.00)	0.17	-0.00	(-0.00, 0.00)	0.38
Neovascu-larization	Yes	0.04	(-0.10, 0.19)	0.42	0.08	(-0.08, 0.23)	0.22
	No	Reference			Reference		
Knee-to-wall		0.00	(0.00, 0.01)	0.24	0.00	(-0.01, 0.01)	0.79
Heel raise		-0.00	(-0.00, 0.00)	0.59	-0.00	(-0.00, 0.00)	0.87

^**†**^ indicates a significant difference (p < 0.05).

Note that the estimates of “0.00” and “-0.00” were rounded and had valid values behind the second decimal position.

Abbreviations: BMI = body mass index; Waist-to-hip = waist-to-hip ratio; Year running = years of running; Current pain = presence of current Achilles tendon pain; Pain history = history of Achilles tendon pain; VISA-A = Victorian Institute of Sport Assessment—Achilles; CSA = cross-sectional area; Knee-to-wall = knee-to-wall composite dorsiflexion; Heel raise = heel raise endurance.

## Discussion

The overall purpose of this study was to identify factors related to sonography-based, intra-tendinous morphological changes in the Achilles tendon in runners. Our findings suggest that male sex and the presence of current Achilles tendon pain were possible factors related to intra-tendinous morphological alterations in the Achilles tendon of runners. When all predictors were considered, male sex remained to be a predictor of low PSFR of the Achilles tendon of runners.

The findings of this study were in accordance with existing literature suggesting that male sex is associated with degenerations of the Achilles tendon[17, 18]. Specifically, our study suggests that male sex is related to a reduction of PSFR value of 0.08–0.10 mm^-1^. The hormone milieu in males has been suggested to influence connective tissue metabolism[31]. Specifically, without the estradiol-inhibiting effect on collagen synthesis, it has been shown that males have a higher collagen synthesis rate than females at rest and after exercise[31]. Despite the higher rate of collagen synthesis, the re-alignment of collagen fibers remains to be a time-consuming process and highly relies on proper mechanical loading (i.e., controlled tensile loading within the toe/linear regions of the stress-strain curve) applied to the tendon[32]. Achilles tendon loading during running has been found to be significantly greater in male runners in comparison to females[17]. The presumably higher collagen metabolic rate and excessive tendon loading may, in part, contribute to disorganization of tendon collagen fibers (i.e., lower PSFR) observed in the male runners of the current study. Additionally, the presence of current tendon pain reduced the PSFR value of the Achilles tendon by 0.07 mm^-1^, indicating that painful tendons may exhibit signs of tendon degeneration. The link between clinical symptoms and lower PSFR observed in the current study agrees with the findings of Kulig et al.[12] showing that symptomatic individuals exhibit lower PSFR than asymptomatic persons at the Achilles tendon.

In our results, PSFR was not associated with age, anthropometry (BMI and waist-to-hip ratio), years of running, tendon gross morphology, neovascularization, ankle joint range of motion, or plantarflexor muscle performance. The non-significant findings of the generalized linear mixed model may partially be explained by the fact that the majority of this research cohort was relatively young, with normal anthropometric measures and tendon gross morphology. For instance, while obesity (defined as a BMI higher than 30) has been found to be a risk factor of Achilles tendon degeneration[20], only a limited number of our runner participants presented with an excessive BMI and hip-to-waist ratio (Table 1). Furthermore, middle age (41–60 years) has been reported to be associated with increased incidence of Achilles tendinopathy [19]. The average age of our runner participants was 37.9 years, which is younger than the reported at-risk age range. McCrory et al.[21] reported that Achilles tendon injury is related to increased years of running. Given that the participants in our study were relatively young, their running experience (8.8 years on average) is much lower than the at-risk running experience (i.e., nearly 12 years) for Achilles tendon injuries reported by McCrory et al. In our study, only 36 runners were older than 41 years and 22 out of 91 runners had ran more than 12 years. Although a relative lower PSFR was found in older or more experienced cohort, the differences in PSFR were not significant based on t-tests (p > 0.30). Future larger-scale research is required to include more senior/experienced runners for better understanding the association between PSFR and age/running experiences in runners.

It should also be noted that several studies identified contributing factors of Achilles tendon injuries by comparing possible risk factors between two distinct groups (i.e., healthy population and injured group confirmed by altered gross tendon morphology)[22, 23, 25]. From these studies, we have learned that altered plantarflexor muscle performance[22, 23] and decreased dorsiflexion range of motion[25] are factors associated with Achilles tendinopathy. The mean CSA of the Achilles tendon observed in this study cohort was similar to that of healthy controls reported in the literature[3, 33], suggesting that the majority of this cohort did not have evident signs of tendinosis depicted by altered gross tendon morphology. Thus, it was speculated that the aforementioned risk factors may be related to more chronic cases of Achilles tendinopathy.

With respect to the findings of the current study, three major limitations should be recognized. First, not all possible risk factors of Achilles tendinopathy were considered in this study, such as rigid footwear, presence of systemic diseases, poor blood supply, and genetic variants[18]. Second, as we aimed to include runners with different levels of experiences, participants were included if they ran at least 6 miles per week. While a lower mileage may less likely contribute to overload related degeneration of the Achilles tendon, our study provided information on the intra-tendinous morphology in recreational runners with a variety of running experiences. Third, as this work was aimed to determine factors contributing to altered PSFR values, the exact cut-off PSFR value defining the abnormal tendon intra-tendinous morphology remains unknown. Future studies may focus on identifying a diagnostic PSFR value (or range of values) that distinguishes between normal and abnormal intra-tendinous morphology.

In conclusion, our findings suggested that male sex and the presence of current Achilles tendon pain were possible factors related to low PSFR, indicative of intra-tendinous morphological alterations in the Achilles tendon of runners. The data from this work may facilitate the understanding of intra-tendinous morphology in Achilles tendon overuse injuries and influence the treatment and intervention strategies of runners with Achilles tendinopathy.
